# Aging and Allostasis: Using Bayesian Network Analytics to Explore and Evaluate Allostatic Markers in the Context of Aging

**DOI:** 10.3390/diagnostics11020157

**Published:** 2021-01-21

**Authors:** Victor Kallen, Muhammad Tahir, Andrew Bedard, Bart Bongers, Natal van Riel, Nico van Meeteren

**Affiliations:** 1 Department of Microbiology & Systems Biology, Netherlands Organization for Applied Scientific Research (TNO), P.O. Box 360, 3700 AJ Zeist, The Netherlands; rizwan.tno@gmail.com (M.T.); andrew.bedard@tno.nl (A.B.); 2 The Physical Activity and Nutrition INfluences In Ageing (PANINI) Consortium: School of Sport, Exercise and Rehabilitation Sciences, University of Birmingham, Edgbaston, Birmingham B15 2TT, UK; bart.bongers@maastrichtuniversity.nl (B.B.); N.A.W.v.Riel@tue.nl (N.v.R.); meeteren@health-holland.com (N.v.M.); 3 Department of Nutrition and Movement Sciences, NUTRIM School of Nutrition and Translational Research in Metabolism/Department of Epidemiology, Care and Public Health Research Institute (CAPHRI), Faculty of Health, Medicine and Life Sciences, Maastricht University, P.O. Box 616, 6200 MD Maastricht, The Netherlands; 4 Department of Biomedical Engineering, Eindhoven University of Technology, P.O. Box 513, 5300 Eindhoven, The Netherlands; 5 Health~Holland, Top Sector Life Sciences and Health, Wilhelmina van Pruisenweg 104, 2595 AN The Hague, The Netherlands; 6 Erasmus Medical Center, Department of Anesthesiology, P.O. Box 2040, 3000 CA Rotterdam, The Netherlands

**Keywords:** allostasis, allostatic load, allostatic load index, aging, biomarkers, Bayesian belief network, elderly, IL-6, BMI

## Abstract

Allostatic load reflects the cumulative strain on organic functions that may gradually evolve into overt disease. Our aim was to evaluate the allostatic parameters in the context of aging, and identify the parameters that may be suitable for an allostatic load index for elderly people (>60 years). From previously published studies, 11 allostatic (bio)markers could be identified that sustain sufficient variability with aging to capture meaningful changes in health status. Based on reported statistics (prevalence of a biomarker and its associated outcome, and/or an odds/risk ratio relating these two), seven of these could be adopted in a Bayesian Belief Network (BBN), providing the probability of “disturbed” allostasis in any given elder. Additional statistical analyses showed that changes in IL-6 and BMI contributed the most to a “disturbed” allostasis, indicating their prognostic potential in relation to deteriorating health in otherwise generally healthy elderly. In this way, and despite the natural decline in variance that irrevocably alters the prognostic relevance of most allostatic (bio)markers with aging, it appeared possible to outline an allostatic load index specifically for the elderly. The allostatic parameters here identified might consequently be considered a useful basis for future quantitative modelling in the context of (healthy) aging.

## 1. Introduction

Healthy aging is conceptualized by one’s ability to adapt and efficiently respond to endogenous and/or exogenous stressors that occur throughout one’s lifespan (e.g., infections, surgery, and other major life events, like bereavement or divorce) [1,2,3]. The concept of allostasis—“achieving stability through adaptive changes”—describes the dynamic, pluriform, though to some extent orchestrated, neurophysiological and psychophysiological responses that aim to match such demands [4,5,6]. One of the key features of investigating the nature of allostasis is the response of a (pre-defined) range of (bio)markers to health challenges threatening internal homeostasis. These adaptive responses can be either physical, endocrine, immune, neuroendocrine, cardiorespiratory, and/or psychosocial, each of which provides the opportunity to measure and/or monitor changes over time in health status by commonly assessed clinical (bio)markers [1,5,7]. Amongst others, Seeman and Juster and their colleagues [1,5,8] designed multiple methods to define an “Allostatic Load Index” (ALI) as parameter of overall health and resilience by combining and interpreting such markers. Extending on these previous studies, and within the context of the Physical Activity and Nutritional Influence in Aging EU consortium [9], the present study aimed to design an allostatic probability model indicating health status in specifically the elderly.

In their landmark paper, Juster et al. [1] published an extensive overview of parameters that are repeatedly used in studies describing allostasis and allostatic load. The latter is defined as the cumulative strain, or “wear and tear” of severe or chronic stress on one’s health. Quite fundamentally, however, there are some challenges associated with the concept of allostasis, specifically in (relatively high-risk) populations like the elderly. Primarily, the gradual and progressive deterioration of health might be considered a fundamental factor in “aging” [10]. Although this process might initially be reflected in subtle—allostatic—disturbances, these disturbances may over time evolve into full-blown syndromes like dementia, metabolic syndrome, diabetes, chronic infections, cardiovascular disease, and/or cancer [11,12,13,14,15,16]. 

The process of aging typically implies a funnel of steadily declining (neuro)endocrine, physiological, and psychophysiological variance components: with time, the natural variance in independent (bio)markers decreases significantly, and consequently, the cumulative overall variance (e.g., captured in constructs like Allostatic Load), reflecting a steadily decrease of the adaptive capability of an individual. This process will inevitably lead to critical transitions in health status (towards (chronic) disease and/or mortality), that might likely, though not yet proven, follow specific mathematical trajectories of decay [17], and is recently even linked to (epi-)genetic effects [18,19,20]. However, this would render biomarkers typically used to define an Allostatic Load Index less useful for application in elderly populations, as sufficient systemic variance to effectively monitor changes in health status declines over time. Dopamine markers (tyrosine hydroxylase, dopamine, and various associated receptors), for example, culminate in a 40–50% reduction between 18 and 88 years; while aldosterone concentrations can be reduced as much as 50% by the age of 70; and testosterone concentrations decline on average by 25% between 25 and 75 years of age in healthy aging men [21,22,23]. Nevertheless, the concepts of allostasis, allostatic load, and ALI appear to be applied quite liberally in the emerging context of personalized medicine and in relation to aging [10,24,25]. 

Consequently, previously published studies do not necessarily incorporate all identified—allostatic—parameters (typically being 26, as firstly advocated by Juster [1]) in reported subsets and/or different versions of Allostatic Load Indices [8,14,26]. Only a selected subset of these markers was, for example, used in a study to define the 25th and 75th percentile in burn-out and exhaustion profiles in generally healthy adults [8]. Studies like these underline the general applicability of the overall ALI design, although they seem to hinder standardization and consequently the interpretation of results: the incorporated parameters are typically selected pragmatically, using a selection that seems to be based on the parameters that are routinely assessed within the collaborating institute(s) [14,26]. 

Finally, in the elderly, major life events (e.g., stroke, surgery, loss) might have a significant impact on their already relatively ‘frail’ health status, with full ‘allostatic’ recovery becoming ever less likely when aging progresses. Consequently, accurately monitoring even subtle changes in health status, for example anticipating and/or in response to major life events, specifically in populations with a natural decline in adaptive capability, might be considered highly relevant, as illustrated by Thomas and colleagues [27]. To tackle this challenge, the concept of ALI might provide a useful starting point for the development of a more or less standardized ALI in the elderly (ALI^E^). 

For these reasons, the present paper aimed to outline a model quantifying the likelihood of allostatic state being “disturbed”, or an allostatic load being built up, in any given elder. This implies that the resulting coefficient (ALI^E^) should be able to capture meaningful changes in the health status of specifically elderly, and/or adequately quantify inter-individual differences in health prognosis. Consequently, we hypothesize that only those allostatic markers with sufficient natural variance in aging populations provide a significant contribution to such a coefficient and should be adopted. 

## 2. Materials and Methods 

### 2.1. Search Strategy

To design a concept of Allostatic Load that can be usefully applied to the elderly, the shortlist of allostatic markers published by Juster [1] was used as reference. Using these allostatic markers, in combination with other potentially relevant terms (“major life event”, “trauma”, “surgery”, “stress”, and “ageing”/”aging”) a comprehensive literature search was performed on PubMed. For example, “Ageing”/“Aging” AND “Cortisol”, or “Stress” AND “BMI”. These terms were combined exhaustively until all pairs of terms had been searched. 

### 2.2. Study Selection

A manual search of abstracts from the collected studies was performed to assess the relevance of the studies found for the present aim. After a first evaluation and selection of appropriate studies, an additional and extensive search in PubMed and Scopus was conducted for those allostatic markers that were not yet, or not yet satisfactory, covered. For each defined relation in the eventual model, only the statistics of the methodologically strongest study were used (e.g., based on sample size, statistical method, and/or accurate reporting of results). This means that due to methodological (Bayesian) restrictions, only a small subset of the initial library could be incorporated (i.e., no additional efforts were conducted to calculate meta-data from multiple studies on specific variables).

For every included study, the following criteria were used to establish eligibility for our modeling efforts: studies should include a (sub)sample of subjects >60 years of age; should incorporate at least one of the 26 allostatic parameters as published by Juster [1]; and should report accurate statistics for Bayesian modelling methods (prevalence of states prior and posterior, and risk ratio (RR) or odds ratio (OR) related to the state of the defined parameter following disease onset). For these reasons, the studied allostatic (bio)markers should be related to the development over time of well-defined health outcomes, for example type II diabetes, stroke, or long-term mortality. The identified syndromes and/or disease types were imputed as separate nodes in the probabilistic model as they were considered to contribute significantly to “disturbed allostasis”, or the retrospective conformation of an “allostatic load” having been building up.

Exclusion criteria were: the defined allostatic (bio)marker significantly declines with aging, and consequently the expected proportional variance factor in the model rapidly decreases in the elderly (becoming “rigid”, non-responsive, or even absent) [28]; outcome measures associated with neurodegenerative disorders like dementia (e.g., Alzheimer’s disorder), schizophrenia, and the like, as they are considered to be fundamentally pathological progressive syndromes; or no appropriate statistics were provided in published studies to implement them in the proposed Bayesian model.

### 2.3. Bayesian Belief Networks 

**Figure 1:**
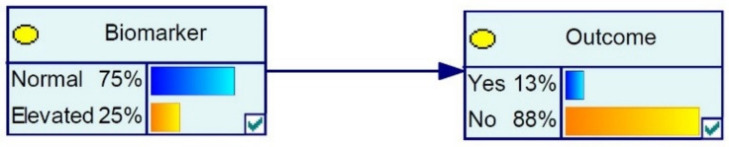
BBN with two nodes in an unobserved state, with Outcome being conditionally dependent on Biomarker state. Using GeNIe software Version 2.3 Graphical User Interface (GUI), the BBN is built up relationship by relationship and can immediately produce predictions based on observed or unobserved states using built-in functionality.

A Bayesian belief network (BBN) is a probabilistic graphical model that represents statistical relationships among variables [29,30]. To establish the weight or contribution of each selected allostatic marker on the likelihood of a disturbed health status of an elderly individual (typically defined as allostatic load), a discrete BBN approach was applied. The Bayesian network approach appears the most suitable modeling method, as the outcomes can provide probabilities, or the likelihood that an allostatic load is present in any given individual. This provides the additional opportunity to monitor whether allostatic states are changing: either within subjects (e.g., over time) or between subjects with other combinations of the state of incorporated allostatic biomarkers. In the present study, the relationships between the events in the network were consequently defined as discrete conditional probability distribution, thus every event (e.g., “stroke recovery”) is a discrete state (either “yes” or “no”) of any variable it is associated with (e.g., C-Reactive Protein: “normal” versus “elevated”). Such a relationship of a biomarker to an associated outcome, each with two discrete states results in a network of two nodes connected by an arc, with four possible discrete sets of evidence. The direction of the arc connecting nodes indicates the dependency of the relationship between the biomarker and outcome, which is defined in the literature from which the conditional distributions are taken (Figure 1). The fundamental advantage of this approach is that the conditional probability distributions can be constructed using commonly reported statistics, in this case RR, OR, interquartile range, and prevalence. This differs from other common AI models in that it does not require data for training or testing, as the conditional probability distributions are entirely self-contained and are deterministic with respect to a complete set of evidence for the network. In this way, it is possible to construct a probabilistic prototype graphical model that represents statistical relationships among variables based on their conditional dependencies, with a disturbed allostatic state as the overall dependent outcome. 

Such a model can be constructed node by node using the component marginal and conditional probabilities by utilizing the generalized form of Bayes Theorem:

$$P(A_{i}|B)\;=\frac{\;P\left(A_{i}\right)P(B|A_{i})}{\sum P\left(A_{i}\right)P(B|A_{i})}$$

where $P\left(A_{i}\right)$, $P\left(B\right),$ and $P\left(A_{i}|B\right)$ are the probabilities of event $A_{i}$, B, and $A_{i}$ given event B, respectively. Bayes Theorem allows us to instantaneously update the joint probability distribution representing the outcome obtained from the model whenever new evidence is provided, a functionality that is handled automatically via the GeNIe software Graphical User Interface (GUI) (see Figure 1).

### 2.4. Modeling

The approach used to build the BBN can be seen as an additive or “bottom up” in that, instead of building a Bayesian network that fits collected data, a network was built one node at a time from published data, including prevalence and distributions of both biomarkers and related medical outcomes. This was made possible through the use of the GeNIe Version 2.3 (https://www.bayesfusion.com/), which through a Graphical User Interface (GUI) allows for the creation of the network structure (nodes and arcs) as well as populating those nodes with conditional probabilities between nodes represented by prevalence and conditional distributions. Due to the sparse amount of published data on the biomarker components of allostatic load in elderly, a stepped model approach was chosen:

Model 1, a BBN model: using published data collected from elderly populations (approximately >60 years of age), where proper statistics were available, along with well-defined cutoffs for biomarkers.

Model 2, an extended version of model 1: considering any published data relating the relevant allostatic markers to each other, but specifically in the context of aged populations. This implies the exclusion of particular parameters that either decline or increase to an extent that their natural variance does not contribute (any more) to the effective monitoring of meaningful changes in health status in elderly. For example over time or circumstances (e.g., due to aging or overt disease states). 

Overall, this resulted in a shortlist of allostatic biomarkers that should be valid and applicable, e.g., in more conservative formats of Allostatic Load Indices, in the elderly.

## 3. Results

**Figure 2:**
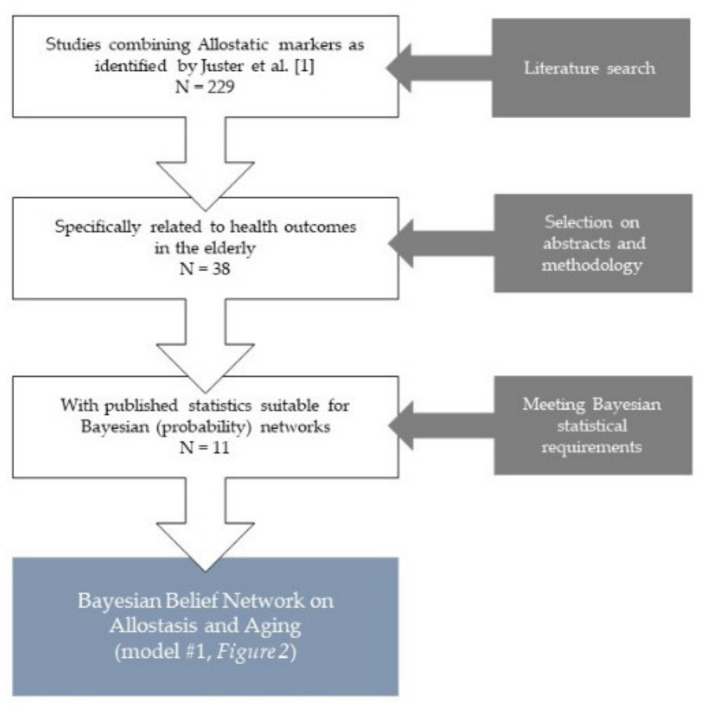
The results of our literature search to allostatic (bio)markers related to health status of well-defined disease states in the elderly (see Table 1).

Our search strategy resulted in 38 studies covering elderly populations, of which 11 met the Bayesian requirements of published prevalence of deviated values of the relevant (allostatic) (bio)markers directly related to well defined outcome states (like specific diseases, or mortality within a defined period of time) (see Figure 2 and Table 1). Typically, the studies could be clustered in a mathematical and/or in a theoretical framework: (1) studies providing the required statistics in elderly populations to design a Bayesian (probability) network model (model #1, mathematical); and (2) studies providing insights in relevant relations (e.g., by means of correlations and/or regression coefficients) within the concept of allostasis; however, no appropriate statistics were published to incorporate them in the Bayesian model (model #2, theoretical). Additionally, due to age-related decline in variance, some established allostatic markers (e.g., aldosterone, albumin) were regarded too rigid to provide useful information on changes in health status in elderly populations. These were consequently excluded from both models. See Table 1 for an overview. 

### 3.1. Model 1: A Probabilistic Allostatic Model in Elderly

**Figure 3:**
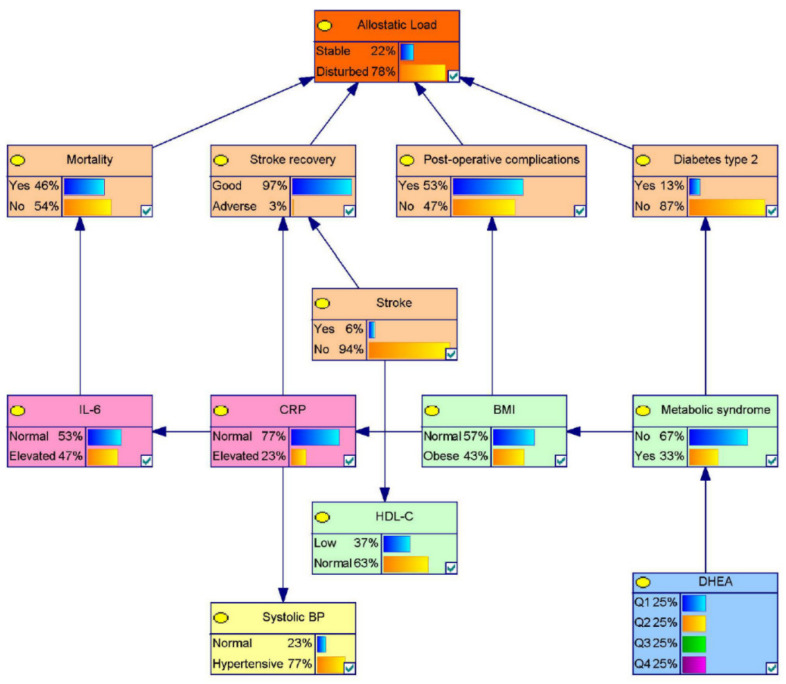
A graphical representation of the probability—allostatic—model for the elderly (>60 years), eventually based on 11 independently published statistical sources (see Table 2). Showing prognostic probabilities on the eventual “overall” outcome, being defined as “allostatic load” (likelihood for progressively deteriorating health). Percentages represent the probability of observing the given level or outcome, in this case, when all states are unobserved. Generated in GeNIe Version 2.3. Note: BMI = Body mass index; BP = Blood pressure; CRP = C-reactive protein; DHEA = Dehydroepiandrosterone; HDL-C = High-density lipoprotein cholesterol; IL-6 = Interleukin-6.

The 11 published studies covering any type of allostatic markers in the elderly, providing statistics to contribute to the proposed Bayesian model, are represented in Table 1. The reported outcome states are typically related to metabolic syndromes, type II diabetes, postoperative complications, general mortality within a defined timeframe (typically six to 10 years), stroke, and stroke recovery (during the six weeks following the incident itself) (see Figure 3). Table 2 provides the underlying prevalence and distribution statistics (with appropriate references), and Table 3 provides the derived probabilities, initially for the identified medical conditions or states, though with the cumulative overall “outcome” being (re)defined as “allostatic load” (see Figure 3). “Stable” indicates that an individual’s current health state does not predispose him or her to a higher risk of negative health outcomes, whereas “disturbed” means that the instantaneous risk of developing adverse health conditions is comparatively higher due to deviating values of the (bio)markers included in the equation.

#### Sensitivity Analysis for the Allostasis Model for Elderly

A sensitivity analysis was performed using built-in functionality in GeNIe Version 2.3 to determine the influence of the individual nodes in our BBN. Sensitivity analysis allowed us to determine the influence of observing the states of specific nodes, which implies the proportional importance of the node in relation to the overall outcome that was defined as allostatic load.

**Figure 4:**
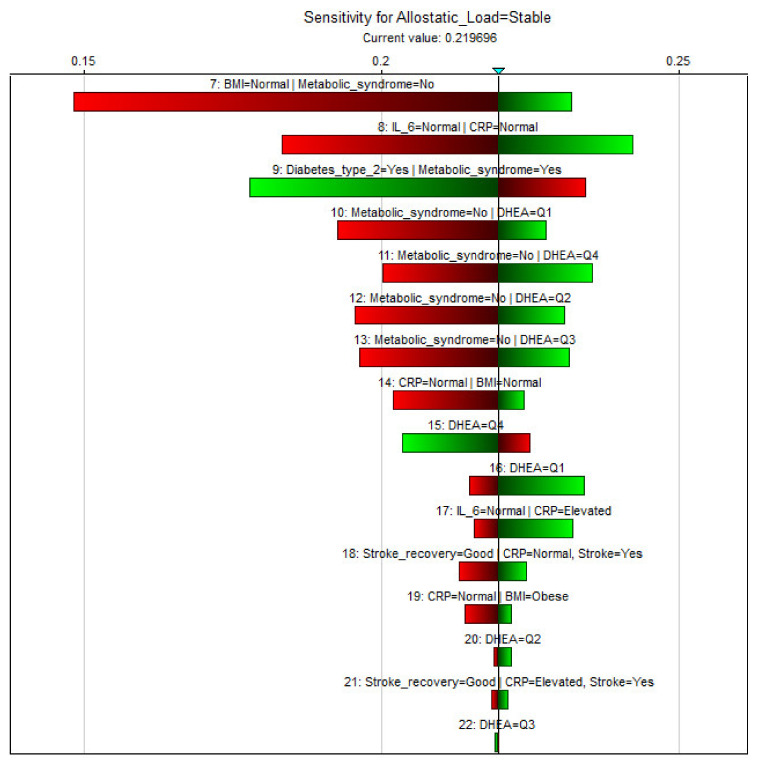
Tornado plot of sensitivity analysis performed in GeNIe software. Label represents observed state, length of color bar represents magnitude of influence on model outcome achieved by changing specified state, with the green side of the bar representing the resulting probability that $P\left(Allostatic\_Load\;=\;Stable\right)$ for the states represented, and the red side representing the resulting probability after reversing the observed state (e.g., Metabolic Syndrome absent/present). For example, following this model, the probability of a stable/undisturbed allostatic load given a normal BMI and the established absence of Metabolic Syndrome, with no information on any of the other parameters, would be 29%; while with BMI = obese, though yet no diagnosed Metabolic syndrome, this likelihood would decrease to 16%. Note: BMI = Body mass index; CRP = C-reactive protein; DHEA = Dehydroepiandrosterone; IL-6 = Interleukin-6.

In Figure 4, a visual representation of specific states of nodes on the outcome of the model being stable is presented. Stable means a (on average) static and low-to-absent allostatic load, indicating the likelihood of a generally good overall health, given the defined states of the associated parameters. The influence of the various medical outcomes and conditions were ignored, since they are seen as intermediate outcomes of our model and as such should exert a disproportionately large influence on the model. Following from previous published statistics the most influential parameters on allostatic load in the elderly appear to be, in order of statistical significance, Body Mass Index (BMI) and interleukin-6 (IL-6). This parametric behavior can also be seen in Table 3: of all investigated parameters, observed changes in BMI and IL-6 concentrations seem to be strongest related to the probable development of allostatic load.

### 3.2. Model 2: An Extended Framework Covering Allostasis in Elderly

**Figure 5:**
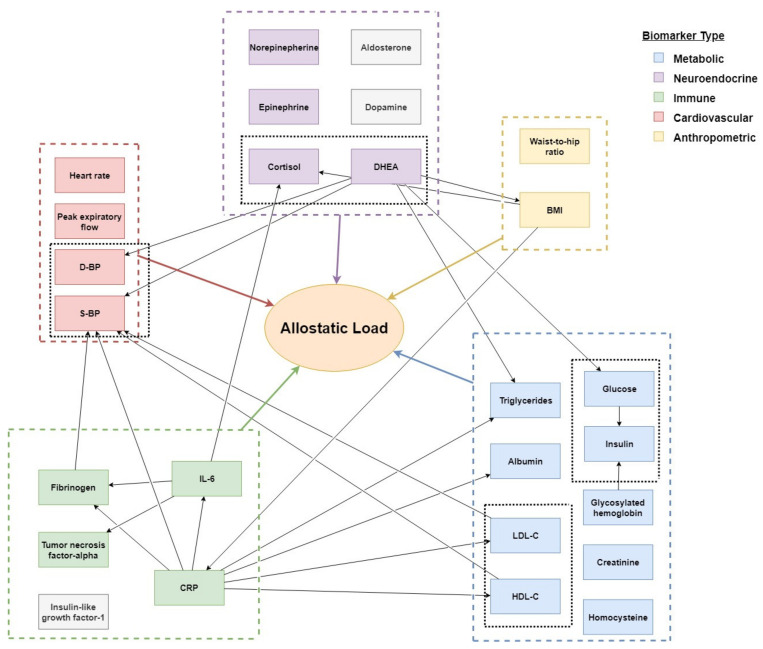
Although not all found studies provided data that could be used in a Bayesian network, based on the collected body of literature an additional (theoretical) model of allostasis in the elderly could nevertheless be constructed (see Table 4 as well). It shows that the biomarker clusters proposed by Juster et al. [1] seem to be valid in elder populations, while for example the cortisol/DHEA ratio [66,67] gains more prominence (as compared to the mathematical (Bayesian) model). The latter finding may call for additional studies to further disclose the actual contribution of such biomarkers in the overall health of elderly.

Our effort to develop a statistically sound (Bayesian) probability model to quantify the probability of disturbed allostasis in elderly (aged >60 years) provided a well-founded framework (model #1, see Figure 3 and Table 2 and Table 3), but yet incorporated only a very small subset of pre-identified allostatic markers. Apart from a significant decrease in variability along with aging, the justification to exclude other established (bio)markers (like high-density lipoprotein (HDL)- cholesterol, and/or blood pressure (BP)), were typically the strict requirements of published statistics for Bayesian modeling. However, other statistically significant relationships found between allostatic (bio)markers and the health status of elderly are summarized in Figure 5 and Table 4. 

## 4. Discussion

The present study aimed to quantify allostatic load on a continuous, probabilistic scale, specifically in elderly. Although it appears to be among the first of its kind, the results presented here are based on the allostatic shortlist published by Juster [1], and by previous efforts to outline a composite—allostatic—assessment, for example by Seeman [5] and Freire [25]. Our study aimed to extend these by focusing both on the elderly and providing a probabilistic model for disturbed allostasis as a required step in the development of clinical decision-making applications. The relevance of empirically validated, reliable, though efficient health assessment protocols, for example for specific high-risk populations like the elderly, has clearly been emphasized by the present Covid-19 pandemic. To live up to their potential (e.g., under pandemic conditions) such applications should consequently be relatively easy to apply on an extensive scale, for example in living environments and communities. 

Because Bayesian networks offer the greatest amount of flexibility given the type of data we expected to find in literature, this method was adopted to investigate and explore previously defined allostatic markers in elderly. An additional and significant advantage of this methodology is that it provides prognostic outcomes for individuals as output, simplifying the translation of the results in clinical decision-making applications, meaning that when the data of any given individual is implemented (in the present format typically being binary: low/high versus normal; or in case of DHEA providing quartile scores), the model will automatically provide a personalized probability for a potentially present allostatic load. 

The study’s aim and methodology provided two significant challenges. Firstly, the adopted methodology prescribed strict statistical/data requirements, implying that only a few allostatic markers as defined by Juster [1] could be adopted in our probabilistic model, as useful, published data (especially in elderly samples) appeared to be rare. Nevertheless, all defined allostatic domains, being neuroendocrine, immune, metabolic, cardiorespiratory, and anthropometric, were represented by at least one parameter in model 1 (see Figure 3), therefore supporting the validity of this “proof of concept”.

Secondly, the natural rate of degradation in variance of many allostatic (bio)markers may actually diminish their relevance in elderly populations. This finding may very well be closely associated with the abovementioned methodological challenge of notably few studies published on specific allostatic (bio)markers in the elderly. This may be due to an either gradual or abrupt, though progressive and significant, reduction in the proportional contribution of quite some biomarkers on changing health status in aging [22,33,35]. 

### 4.1. The Concept of Allostasis in Aging Populations

Aldosterone, for example, appears to slowly decrease with aging [22], although some individuals develop hyperaldosteronism, which might be due to increased ACTH stimulation of the adrenal glands, likely caused by genetic predispositions [69]. Serum concentrations of dehydroepiandrosterone (DHEA) and its sulfate (DHEA-S) have been reported to peak in young adulthood, followed by a steady decline over the following decades (between 40 and 80 years of age) by approximately 60%. Combined with increasing levels of cortisol [33,34,70], and small, but significant, decreases in the serum concentrations of progesterone, it is suggested that adrenal functioning is particularly affected by aging [71], and may actually constitute an alternative and composite allostatic (sub)structure (e.g., cortisol–DHEA ratio) in aging populations. Importantly, the steady increase in cortisol–DHEA ratio with aging is suggested to relate to immune deficits and in this way to the prevalence of infectious diseases [66,67]. For this reason, the contribution of the cortisol–DHEA ratio in quantifying allostatic load in elderly, despite the declining singular contribution of especially DHEA, may nevertheless appear relevant. Many other associated (bio)markers interact either within the specified domains of allostasis, or between them. C-reactive protein, for instance, has been suggested to interact with both metabolic and cardiorespiratory processes, apart from its established status as an immune marker (see Table 1 and Figure 5). Likewise, IL-6 and fibrinogen interact with neuroendocrine and cardiovascular processes, respectively. Representative examples on how such relationships with allostatic load can be captured in computational models are earlier presented [72,73]. 

It seems valid, however, to conclude that with aging, the contribution of most allostatic (bio)markers on health irrevocably changes and in some cases might even diminish, as measurable concentrations may progressively decline and become insignificant, a process that might be a very fundamental component of aging in itself. Not necessarily surprisingly, the present study provides ample evidence that their potential as health-related parameters, either independently or incorporated in any kind of allostatic index, seems to gradually decline. This means that from a normative perspective, the allostatic load will steadily increase with age, whilst the standardized variance over this cumulative load is likely to substantially decrease. This is a process with significant inter-individual differences in progression, which are likely directly associated with exogeneous factors like exposure to infections, lifestyle, medical procedures, major life events, and/or psychological strain. It seems a valid conclusion that studies unraveling such allostatic dynamics associated with generally good or slowly declining health in elderly remain relevant.

Based on the present findings, in the elderly, allostatic markers seem to be categorized in three classes: (1) markers that gradually decline with aging, with insufficient variance left in elderly to adequately capture changes in health status over time (like aldosterone); (2) allostatic markers that maintain significant variance to show an adaptive response to repetitive or severe stress (e.g., BMI and some immune markers), likely associated with allostatic processes and overall health status; (3) markers with a yet highly significant variability and reactivity in response to immediate stressors (e.g., cortisol, epinephrine, and creatinine), being more or less representative for the sustained systemic flexibility and variability [28], and in this way potentially contributing to the quantification of allostasis in elderly populations. The last two categories obviously are, at least theoretically, still useful for monitoring health status and/or impact of and recovery from significant events or health threats in the elderly (see Table 4).

Importantly, the present results are supported by a recent study of Freire et al. who used a selection of allostatic (bio)markers in the elderly that showed a remarkable resemblance to the results presented here (Table 3 as compared to Table 4 in the present study). Although a conventional method was used to calculate Allostatic Load (compared to the here-used probabilistic approach), their findings may consequently be considered a relevant cross-validation of the here-identified allostatic parameters for elderly [25]. 

### 4.2. Limitations

Due to the aforementioned statistical requirements, a mathematically robust but yet condensed probabilistic allostasis model could be constructed. However, as acknowledged by the authors, from an allostasis perspective, it seems far from complete. Apart from the previously mentioned restrictions, it implies that allostatic parameters that are directly associated with very specific disorders are less useful in this context, as their variance contribution is disproportionately mediated and/or moderated by the presence or absence of the associated disorder. For example, due to its significant relation with dementias specifically, dopamine was excluded from the models. Consequently, in its current form, the two models outlined here represent the potentially changing health status of (yet) generally healthy elderly people. 

Secondly, a BBN model can only go as far as to provide the instantaneous probability of an individual suffering from a disturbed allostatic load based on the biomarker data collected in references to the overall (population based) statistics. However, it does not provide predictive power regarding causation, and only when frequently assessed might provide some information on relevant changes over time. To capture “allostatic dynamics” on an individual level, more advanced types of mathematical/data driven approaches seem essential. For example machine learning methods using dynamic (time-series) data based on repeated measures designs, with relatively high sampling frequencies of the relevant (bio)markers. Such exercises might be of significant importance to progress in the personalized medicine of aging, although they are not found in the literature so far. In this perspective, it is worthwhile to note that only few previous efforts have been made to use Bayesian modeling in the context of allostasis, with the study of Stephan [74] a notable exception. This might be considered somewhat surprising in the context of “precision medicine”, because accurate probability and/or prognostic models seem to be essential to designing effective personalized monitoring and prevention strategies, not least so in the elderly. 

The identified categories of variance components in relation to a developing allostatic load in elderly (rigid or absent; adaptive variance related to mid-term changes in health status; or responsive variance in an immediate reaction to stressors) underline another tangible matter: disparate phase patterns in response to exogenous stressors in the elderly. Whilst some parameters rapidly respond and recover (within seconds to minutes: “responsive”), others might become disturbed more extensively with considerably longer recovery trajectories over time. Consequently, when allostatic load is considered as a more continuous scale, it may be concluded that the overall “allostatic” variance in any given individual contains quite different variance ranges, both in time and extent, originating from the heterogenous combination of incorporated parameters. Patterns of multilevel frequencies of variance may very well follow general mathematical outlines for early warning signals for upcoming critical transitions, as addressed above [17]. In the context of personalized medicine, more advanced analytics of the different variance components might for that reason be highly relevant to progress from the typical instantaneous snapshot provided with the presently applied frameworks. 

Finally, of course other frameworks and/or parameters could be considered to capture (challenged) health status in elderly. Al Saedi [75], for example, provided a very comprehensive overview of biomarkers associated with frailty in aging, including parameters that have been extensively utilized in aging literature (e.g., muscle mass, grip strength, and metabolites associated with oxidation processes). However, since the theory of allostasis is one of the few that provides a validated and comprehensive framework for the assessment of health status, and based on the general consensus on the relevance of the parameters published by Juster [1], it was decided to start from there. 

### 4.3. Conclusions

Apart from other mathematical approaches to model and quantify Allostatic Load [73], the present study provided one of the first probabilistic allostatic models, notably in the elderly. Although the natural decline in variance irrevocably alters the prognostic relevance of most allostatic (bio)markers, in this way, it appeared possible to outline an allostatic load index specifically modified for the elderly (ALI^E^). Together with the 11 allostatic parameters identified here, this “proof of concept” may therefore be considered as a useful basis for future quantitative modeling in healthy aging, not least because allostasis and allostatic load are often referred to in studies of the elderly, but primarily as a theoretical concept. Consequently, future efforts may focus on further developing probabilistic and/or prognostic models in the elderly based on the concept of allostasis, and in this way refine their applicability in the context of personalized medicine. 

## Figures and Tables

**Table 1:** Allostatic parameters identified by Juster [1], with in- and exclusion criteria for adoption in either the probability model 1 (see Figure 2 and Figure 3), or the more theoretical framework (see Figure 5), both aiming to capture health status in specifically elderly (aged >60 years).

No	Allostatic Biomarker	Included in Models 1 and 2	Included in Model 2 (√), or Excluded (X)	Effects of Aging	Associated Health Outcomes	References
	**Neuroendocrine**					
1	Cortisol		√	Stable or increasing; remains highly responsive	Depressed mood; anxiety; hostility; and adiposity.	Ai et al., 2014 [31] Jackson et al., 2017 [32] Heaney et al., 2014 [33] Peeters et al., 2008 [34]
2	Dehydroepiandrosterone (DHEA)	√		Significant decline with aging; age 70–80 years ~20% compared to those aged 20–30 years	Musculoskeletal disorders; Cognitive disorders; Mood disorders; Cardiovascular disease; Sexual functioning; Menopause symptoms.	Heaney et al., 2014 [33] Samaras et al., 2013 [35] Baulieu et al., 2000 [36]
3	Epinephrine (EPI)		√	Higher basal plasma concentrations and higher responses to acute stressor.		Pascualya et al., 1999 [37]
4	Norepinephrine (NE)		√	Higher basal plasma concentrations and higher responses to acute stressor.		Pascualya et al., 1999 [37]
5	Dopamine		X	Averaged 6–10% loss per decade, resulting in 40–50% loss between 18 and 88 years.	Parkinson; dementia.	Bäckman et al., 2006 [21] Reeves et al., 2002 [38]
6	Aldosterone		X	Reduced (plasma) concentrations up to 50% at age 70 years.	Associated with progress of coronary artery disease.	Bauer, 1993 [22]
	**Immune**					
7	Interleukin-6	√		Age-related pro-inflammatory state due to intrinsic dysregulation of the immune system.	Inflammation; morbidity; cardiovascular disease; diabetes mellitus, sarcopenia, dementia; depressed mood; anxiety; and hostility.	Ai et al., 2014 [31] Li et al., 2017 [39]
8	Tumor necrosis factor-alpha (TNF-α)		√	Increasing circulating levels of TNF-α.	Atherosclerosis.	Bruunsgaard et al., 2000 [40]
9	C-reactive protein or high sensitivity C-reactive protein	√		General risk factor associated with aging-related diseases.	Cardiovascular disease; hypertension; diabetes mellitus; kidney disease; atherosclerosis; depressed mood; anxiety; and hostility.	Ai et al., 2014 [31] Bruunsgaard et al., 2000 [40] Tang et al., 2017 [41]
10	Insulin like growth factor-1 (IGF-1)		X	Declining (up to 60%).	Bone loss; and osteoporotic fractures.	Seck et al., 1998 [42] Garnero et al., 2000 [43]
11	Fibrinogen		√	Increase in 25 mq/dl per decade.	Cardio- and cerebrovascular disease.	Hager et al., 1994 [44]
	**Metabolic**					
12	HDL-cholesterol	√		Seems stable.	Cardiovascular diseases.	Holzer et al., 2013 [45]
13	LDL-cholesterol		√	Increases with aging.	Cardiovascular diseases.	Holzer et al., 2013 [45]
14	Triglycerides		X	Increases with aging	Cardiovascular diseases.	Holzer et al., 2013 [45]
15	Glycosylated hemoglobin (HbA1c)		√	Increases with aging.	Cardiovascular & ischemic heart diseases; and diabetes.	Dubowitz et al., 2014 [46]
16	Glucose		√	Glucose hemostasis gets disturbed with aging.	Primarily, though not exclusively, diabetes.	Van den Beld et al., 2018 [23]
17	Insulin		√	Decreases with aging.	Diabetes and associated health problems.	Van den Beld et al., 2018 [23]
18	Albumin		X	Decreases with aging.	Loss of appendicular muscle mass; sarcopenia.	Visser et al., 2005 [47]
19	Creatinine		√	Decreases with aging.	Renal function.	Friedlander et al., 2014 [48]
20	Homocysteine		X	Increases with aging.	Alzheimer’s disease; lower cognitive performance; stroke.	Pařízková et al., 2017 [49] Matsui et al., 2001 [50]
	**Cardiovascular & Respiratory**					
21	Systolic blood pressure *	√		High incidence of hypertension (>140 mm Hg) in elderly.	Hypertension; cardiovascular problems; cerebrovascular morbidity; mortality.	Rigaud et al., 2001 [51]
22	Diastolic blood pressure *		√	High incidence of hypertension (>90 mm Hg) in elderly.	Hypertension; cardiovascular problems; cerebrovascular morbidity; mortality.	Rigaud et al., (2001) [51]
23	Peak expiratory flow		X	Decreases with aging.	Asthma; COPD; decreased lung function.	Janssens et al., 1999 [52]
24	Heart rate *		√		Cardiovascular morbidity and mortality.	
	**Anthropometric**					
25	Waist-to-hip ratio		√	Increases with aging.	Cardiovascular disease; diabetes mellitus.	Stevens et al., 2010 [53] Huxley et al., 2010 [54] Woo et al., 2002 ** [55]
26	Body mass index (BMI)	√			Many health-related issues, e.g., ischemic heart disease; stroke; and overall mortality.	Prospective Studies Collaboration, 2009 [56] Song et al., 2004 [57] Visscher et al., 2001 [58]

Note: COPD = Chronic obstructive pulmonary disease; DHEA = Dehydroepiandrosterone; HDL-C = High-density lipoprotein cholesterol; LDL-C = Low-density lipoprotein cholesterol. * At rest. ** According to Woo et al. (2002) Waist-to-hip ratio may not be a useful predictor of negative health outcomes in elderly.

**Table 2:** Applied statistics for the probability model (see Figure 3), including references.

Biomarkers or Outcomes	Reported Health Outcome(s) or Interactions	Cutoff Criteria	Reported or Calculated Risk Ratio	References
Dehydroepiandrosterone	Metabolic syndrome	1.44 µmol/L	1	Chen et al., 2010 [59]
		2.31 µmol/L	1.66	
		3.4 µmol/L	1	
		13.5 µmol/L	2.68	
C-reactive protein	Stroke recovery	≤8 mg/L	1.36	Rigaud et al., 2001 [51]
	Interleukin-6		1.97	
	Systolic blood pressure		1.18	
Interleukin-6	Mortality	≤1.8 pg/mL	1.49	Li et al., 2017 [39] Lee et al., 2012 [60]
Body mass index	Postoperative complications	18.5–24.9 kg/m^2^	1.6	Barone et al., 2017 [61] Lee et al., 2012 [60]
	C-reactive protein		1.4	
Stroke	HDL-C	Boolean	1.24	Sacco et al., 2001 [62] Bruckert et al., 2006 [63] Boix et al., 2006 [64]
Metabolic syndrome	Type II diabetes	Boolean	4.42	Sattar et al., 2008 [65]
	Body mass index	Boolean	6.76	Lee et al., 2012 [60]

Note: HDL-C = High-density lipoprotein cholesterol.

**Table 3:** Probabilities of BNN when an outcome is observed, including associated medical conditions in elderly populations. These conditions being intermediate to the eventual overall outcome, being defined as “allostatic load” specified for elderly (>60 years of age) and statistically robust (due to the strictly applied criteria in Bayesian modeling).

		Allostatic Load	
		* Stable	* Disturbed
Mortality within 8 years	Yes	0%	59%
	No	100%	41%
Stroke	Yes	4%	7%
	No	96%	93%
Stroke recovery	Positive	100%	96%
	Adverse	0%	4%
Postoperative complications	Yes	0%	68%
	No	100	32%
Type II diabetes	Yes	0%	16%
	No	100%	84%
Interleukin-6	Elevated	38%	50%
	Normal	62%	50%
C-reactive protein	Elevated	20%	24%
	Normal	80%	76%
Body mass index	Obese	26%	48%
	Normal	74%	52%
Metabolic syndrome	Yes	19%	37%
	No	81%	63%
DHEA	Q1	27%	25%
	Q2	25%	25%
	Q3	25%	25%
	Q4	23%	25%

Note: DHEA = Dehydroepiandrosterone. * Observed model outcome state.

**Table 4:** Allostatic biomarkers with typical yet significant variability in elderly to capture adaptive or immediate reactive responses to exogenous stressors in elderly.

Parameter	Short Comment(s)	References	Reported Age Ranges
Cortisol	Highly responsive up until high age typically use in cortico-DHEA ratio in aging studies	Heaney et al., 2014 [33]	65–88
		Peeters et al., 2008 [34]	65+
Epinephrine	Significant response to an acute stressor: helps a person to cope with physical and emotional stress	Pascualya et al., 1999 [37]	24–26, 69–71, 83–85
Norepinephrine	Insignificant response to acute stressor; however, in elderly this increase is due to increase concentration of plasma concentration and decrease in clearance	Pascualya et al., 1999 [37]	24–26, 69–71, 83–85
Interleukin-6	Due to any dysregulation in the immune systems, circulating interleukin-6 levels are independently associated with greater risk of cardiovascular and all-cause mortality in the general elderly population	Li et al., 2017 [39]	60+
C-reactive protein	Important risk factor in elderly	Ai et al., 2014 [31]	35+
		Bruunsgaard et al., 2000 [40]	19–31, All 81 (n = 130)
		Tang et al., 2017 [41]	Review: 80+
Fibrinogen	Increases by 25 mg/dL/decade: good indicator for variance in an older population	Hager et al., 1994 [44]	23–96
High-density lipoprotein- cholesterol	Good indicator for variance, does not change significantly with aging	Holzer et al., 2013 [45]	25–28, 65–69
Creatinine	24-hr urine creatinine decreases with aging	Friedlander et al., 2014 [48]	<45–65+
Systolic and diastolic blood pressure	Indicator of hypertension; however, variance component is not that much	Rigaud et al. 2001 [68]	60–84

Note: DHEA = Dehydroepiandrosterone.

## Data Availability

No new data were created or analyzed in this study. The sharing of original is consequently not applicable to this article.
